# Hybrid Space Calibrated 3D Network of Diffractive Hyperspectral Optical Imaging Sensor

**DOI:** 10.3390/s24216903

**Published:** 2024-10-28

**Authors:** Hao Fan, Chenxi Li, Bo Gao, Huangrong Xu, Yuwei Chen, Xuming Zhang, Xu Li, Weixing Yu

**Affiliations:** 1 Key Laboratory of Spectral Imaging Technology of Chinese Academy of Sciences, Xi’an Institute of Optics and Precision Mechanics, Xi’an 710119, China; fanhao2022@gmail.com (H.F.); gaobo_101@opt.ac.cn (B.G.); hrxu4221@163.com (H.X.); chenyuwei@opt.ac.cn (Y.C.); 2 Center of Materials Science and Optoelectronics Engineering, University of Chinese Academy of Sciences, Beijing 100049, China; 3 Department of Applied Physics, Hong Kong Polytechnic University, Hongkong 999077, China; apzhang@polyu.edu.hk (X.Z.); xupolyu.li@connect.polyu.hk (X.L.)

**Keywords:** multispectral imaging, diffractive lenses, space calibration, point spread function

## Abstract

Diffractive multispectral optical imaging plays an essential role in optical sensing, which typically suffers from the image blurring problem caused by the spatially variant point spread function. Here, we propose a novel high-quality and efficient hybrid space calibrated 3D network “HSC3D” for spatially variant diffractive multispectral imaging that utilizes the 3D U-Net structure combined with space calibration modules of magnification and rotation effects to achieve high-accuracy eight-channel multispectral restoration. The algorithm combines the advantages of the space calibrated module and U-Net architecture with 3D convolutional layers to improve the image quality of diffractive multispectral imaging without the requirements of complex equipment modifications and large amounts of data. A diffractive multispectral imaging system is established by designing and manufacturing one diffractive lens and four refractive lenses, whose monochromatic aberration is carefully corrected to improve imaging quality. The mean peak signal-to-noise ratio and mean structural similarity index of the reconstructed multispectral images are improved by 3.33 dB and 0.08, respectively, presenting obviously improved image quality compared with a typical Unrolled Network algorithm. The new algorithm with high space calibrated ability and imaging quality has great application potential in diffraction lens spectroscopy and paves a new method for complex practical diffractive multispectral image sensing.

## 1. Introduction

Diffraction multispectral imaging typically uses binary optical diffractive elements which are axially dispersive to realize spectral separation and optical imaging, attracting great interest among researches in optical sensing, such as gas sensing [1], industrial detection [2,3], security sensing [4], and so on. A diffraction lens, the critical optical element of diffraction multispectral imaging, disperses the spectra of incident light, and different spectra are separated at different imaging positions along the optical axis. Since Denise M. Lyons reported the diffractive optic image spectrometer (DOIS) system in 1995 [5], scholars have carried out many studies on diffractive spectral imaging [6,7,8]. In 1999, Michele Hinnrichs et al. proposed a diffractive hyperspectral system for gas sensing with a camcorder-sized prototype and further realized dual-band mid-wavelength infrared/long-wavelength infrared hyperspectral imaging using a single lens and a single dual-band focal plane array in 2003 [9]. Neelam Gupta et al. developed a diffraction imaging spectroscopy system by using a stepper motor on a linear rail, confirming the detection of surface contaminants [2]. Phuong-Ha Cu-Nguyen et al. demonstrated a confocal hyperspectral sensing system utilizing diffractive optical elements and a tunable membrane fluidic lens [10], and further designed the highly compact tunable hyperchromatic lens system at a wavelength range of 450–900 nm [11]. These reported diffractive multispectral imaging systems utilize a stepper motor and a tunable fluidic lens to adjust the position of the image and obtain multispectral images in the visible and infrared spectrum, which shows low spectrum resolution and cannot meet the need of more accurate and more sensitive multispectral diffractive imaging.

With the rapid development of artificial intelligence, many researchers pay attention to the high-accuracy multispectral reconstruction of the diffractive image by introducing AI algorithms to avoid the weaknesses of complex equipment modifications and extensive data processing [12,13,14]. Zhang Ming-qi et al. proposed the so-called inverse filtering restoration algorithm along the path of traditional methods to solve the ill-posed problem of inverse filtering of diffraction spectroscopy imaging [15]. Daniel S. Jeon and coworkers proposed the use of a neural network algorithm in spectral image reconstruction to improve the spectral resolution and imaging quality [16]. F. S. Oktem developed a model-based fast reconstruction algorithm in an extreme ultraviolet regime by combining data-driven and model-driven methods to improve the reconstruction accuracy [17]. Zhao Hai-bo designed a dual-channel visible and near-infrared diffraction computational imaging system that utilizes additional calibration information to improve the spatial resolution of the DOIS for complex imaging scenery [18]. These diffraction spectral algorithms use end-to-end algorithms that are accurate and efficient. However, these methods require substantial real-world data and sophisticated calibration methods to meet the precision demand of neural networks. On the other hand, the hardware improvements of the multispectral diffractive imaging system normally suffer from larger amounts of data, a higher economic expense, and a more complex system due to the utilization of more cameras and spiral diffractive lenses; as a result, they cannot meet the requirements of miniature diffractive multispectral imaging technology.

Furthermore, spatial variation is an important factor in the multispectral diffractive imaging system that involves magnification, rotation, and displacement, which leads to the complex spatially variant point spread function (PSF) dramatically degrading the imaging quality. Michele Hinnrich et al. resampled acquired spectral images with different magnifications, and spectral image construction was then carried out with the same magnification [6]. Qiang Sun et al. added the optical zoom module into the diffractive multispectral imaging system to avoid the magnification effect [19]. These reported works utilize precise PSF estimation, large training datasets, and novel optical structures to enhance the accuracy of multispectral reconstruction in various diffractive multispectral imaging applications. In addition, complex measurements such as shooting, registration, and color correcting are necessary for PSF estimation. Moreover, large training datasets are necessary for hardware improvements to achieve high-accuracy calibrations of targeted images. These traditional methods suffer from complex manual methods and procedures, and again cannot meet the development trend of diffractive multispectral imaging. Thus, how to achieve convenient high-accuracy diffraction spectroscopy imaging that overcomes complex space variation factors and algorithmic limitations in complex sensing is still an open question.

In this paper, we propose a diffractive hyperspectral optical imaging system with a hybrid space calibrated 3D Network “HSC3D” that adopts complex optical sensing with the spatially variant PSF. Our HSC3D algorithm utilizes the 3D U-Net structure with 3D convolutional layers that is constructed by our recently published method [20], which is also combined with the advantages of a space calibration module to improve the imaging quality of reconstructed images. Multispectral data are simulated through the forward process of diffractive optical imaging and are further corrected by using a fabricated calibration module. The diffraction multispectral imaging system is composed of one diffractive lens and four refractive lenses, whose monochromatic aberration is designed and corrected to improve imaging quality. The magnification and rotation variant effects of the measured eight-channel multispectral images are corrected by the spaced calibrated module and further transferred into the 3D U-Net to restructure the multispectral images accurately. The mean peak signal-to-noise ratio (MPSNR) and the mean structure similarity index measure (MSSIM) of the restructured multispectral images are calculated and discussed. The effectiveness of our HSC3D algorithm is also analyzed and compared with a typical Unrolled Network.

This article proceeds as follows. In Section 2, we introduce the hybrid space calibrated 3D Network. In Section 3, we present our established diffractive multispectral imaging system. In Section 4, we analyze the reconstruction of eight-channel multispectral images and compare it with other algorithms. In Section 5, the conclusions of the proposed approach and directions for future work are given.

## 2. Experimental Methods

**Figure 1:**
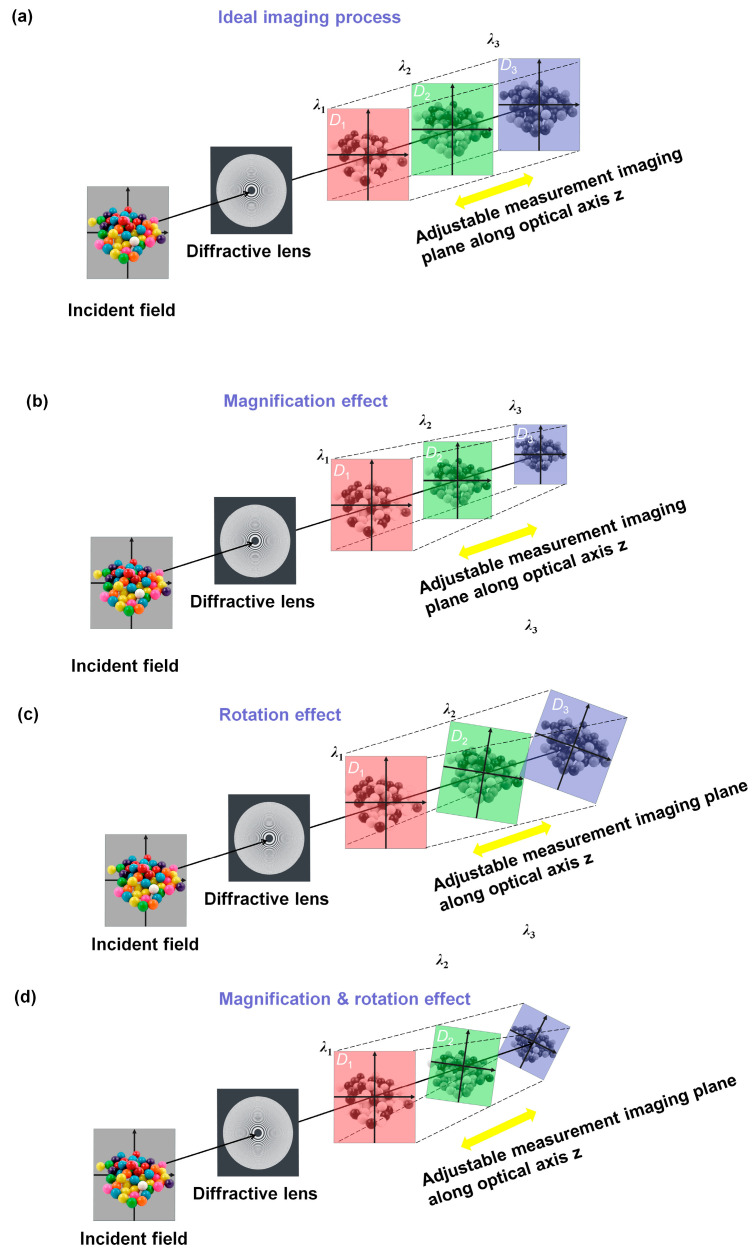
(**a**) The schematic diagram of the ideal imaging process of diffractive multispectral imaging, (**b**) the schematic diagram of the magnification effect of diffractive multispectral imaging, (**c**) the schematic diagram of the rotation effect of diffractive multispectral imaging, and (**d**) the schematic diagram of complex spatial variations combing the magnification and rotation of practical diffractive multispectral imaging, where *λ*_1_, *λ*_2_, and *λ*_3_, denote different wavelengths and satisfy $\lambda_{1}>\lambda_{2}>\lambda_{3}$, and *D*_1_, *D*_2_, and *D*_3_ denote the dimension of diffractive multispectral images at different wavelengths.

Diffraction multispectral imaging typically uses diffractive lenses to disperse light along the optical axis to obtain multispectral information. According to the dispersion properties of the diffractive lenses ($f\left(\lambda \right)=\lambda_{0}f_{0}/\lambda$), the incident light of longer wavelengths is imaged at the front image plane, while the light of shorter wavelengths is imaged in the back image plane [21]. The incident light travels through the diffractive lens and disperses along the optical axis, forming narrowband multispectral images. Multispectral images of different wavelengths can be obtained by moving the image plane by multiple measurements. Figure 1a shows the ideal imaging process of the diffractive multispectral imaging system. When the measurement plane is adjusted to different positions of $z_{1}$, $z_{2}$, and $z_{3}$ along the optical axis, these multispectral images at different wavelength show similar dimensions $\left(D_{1}=D_{2}=D_{3}\right)$ and rotation angles $\left(\theta_{1}=\theta_{2}=\theta_{3}\right)$. Furthermore, the magnification effect of the real multispectral imaging process in Figure 1b presents a smaller dimension $\left(D_{1}<D_{2}<D_{3}\right)$ with a larger wavelength according to the transverse magnification of the system [6]. Figure 1c shows the rotation effect of the real multispectral imaging process, where a different rotation angle $\left(\theta_{1}\neq \theta_{2}\neq \theta_{3}\right)$ emerges among different multispectral images along the optical axis. In addition, as shown in Figure 1d, practical multispectral diffractive imaging presents more complex spatial variations combing magnification and rotation effects, whose multispectral images have different dimensions $\left(D_{1}<D_{2}<D_{3}\right)$ and rotation angles $\left(\theta_{1}\neq \theta_{2}\neq \theta_{3}\right)$. The multispectral imaging information is related to the intensity of each spectral component and can be calculated as follows:

(1) $$g(m,n)=\sum_{\lambda }\sum_{m,n}I(\xi ,\zeta ,\lambda )H(m-\xi ,n-\zeta ,\lambda )+\eta$$

where g(m,n) represents the spectral image, and *m* and *n* denote the center of each pixel in the *x* and *y* direction. I(ξ,ζ,λ) represents the original spectral information, *H* represents the PSF of the diffractive lens, and *η* denotes noise. The PSF of multispectral diffractive imaging can be calculated by using the established method in [20],

(2) $$H(x,y,\lambda )={\left|\frac{1}{\lambda f}e^{\frac{ik}{2f}\left(x^{2}+y^{2}\right)}\iint P(s,t,\lambda )e^{\frac{ik}{2f}\left(s^{2}+t^{2}\right)-\frac{ik}{f}\left(xs+yt\right)}dsdt\right|}^{2},$$

where P⁡(s,t,λ), (*x*, *y*), (*s*, *t*), *f*, *λ* and *k* denote the function of the incident light field, the spatial coordinates in the plane of the image sensor, the spatial coordinates in the plane of the diffractive lens, the distance between the lens and the sensor, the wavelength, and the wave number, respectively. Considering the space-variant effect on practical multispectral diffractive imaging, the calibrated spectral image $y\left(m,n\right)$ can be expressed by

(3) $$y\left(m,n\right)=\sum_{\lambda }\sum_{m,n}H(m-\xi ,n-\zeta ,\lambda )K(\lambda ,z)I(\xi ,\zeta ,\lambda )+\eta ,$$

where the space variation matrix $K\left(\lambda ,z\right)$ is related to the wavelength λ and the focus position z. For a specific wavelength *λ_i_* at a specific position along the optical axis *z_i_*, the space variation matrix K can be expressed as a function of $x_{max}$ and $y_{max}$,

(4) $$K\left(\lambda_{i},z_{i}\right)=f\left(x_{max},y_{max}\right)$$

where $x_{max}$ and $y_{max}$ denote the maximum values of the spatial coordinate in the plane of the image sensor. The dimension *D* of multispectral images for a specific wavelength *l_i_* satisfies ${D=4x}_{max}y_{max}$. Furthermore, the reconstruction of multispectral images can be regarded as a 2D multichannel deconvolution problem and can be formalized as

(5) $$\hat{I}\left(\lambda \right)=argmin_{I}\frac{1}{2}∥y-KHI{∥}_{2}^{2}+\lambda R\left(I\right),$$

where $R\left(I\right)$ is a regularization term that serves as a constrained condition and is used to avoid the overfitting problem.

To obtain the reconstructed multispectral images, the inverse problem of Equation (4) can be separated into the following two steps. Firstly, the measured images are calibrated to remove spatial variations and noise, and can be expressed as

(6) $${\hat{S}}_{1}=argmin_{I}\frac{1}{2}∥y-{KS}_{1}{∥}_{2}^{2}+\lambda R\left(S_{1}\right),$$

where $S_{1}$ represents the environmentally influenced multichannel spectral images. Secondly, the multichannel spectral information is unmixed by means of a multispectral inverse convolutional reconstruction network and can be formalized as

(7) $$\hat{I}\left(\lambda \right)=argmin_{I}\frac{1}{2}∥{\hat{S}}_{1}-HI{∥}_{2}^{2}+\lambda R\left(I\right).$$

In practice, diffractive multispectral imaging commonly presents magnification (Figure 1b) and the rotation effect (Figure 1c), which arises from variable transversal magnification and small vibrations of the imaging system and complex sensing background [22,23].

**Figure 2:**
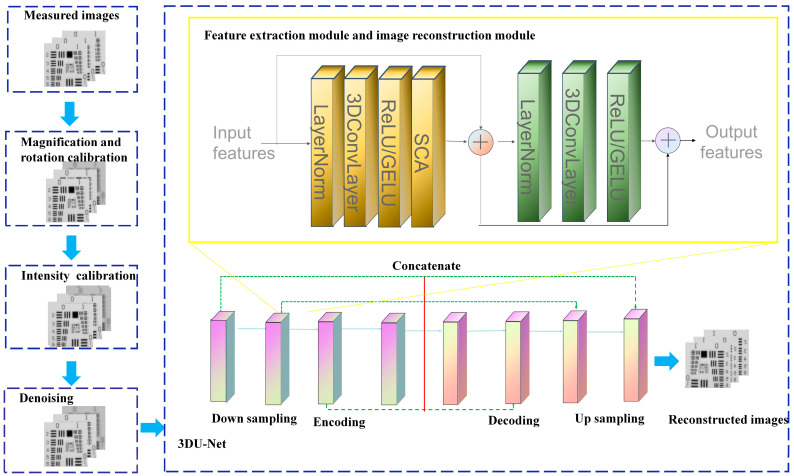
Experimental method of our hybrid space-variant 3D U-Net.

To solve this problem, the 3D network is combined with the space calibration module to achieve high-accuracy multispectral image reconstruction. Figure 2 exhibits the architecture of our HSC3D algorithm that is composed of four critical parts, including magnification and rotation calibration, intensity calibration, denoising, and 3D U-Net reconstruction.

1. Magnification and rotation calibration: The magnification variation *α* of diffractive multispectral imaging at the focal length of different wavelengths is calibrated through simulation of multispectral training data. Also, the random rotation angle difference *β* introduced by the complex imaging progress of the diffractive multispectral system (Figure 1c) is inputted into the data preprocessing step to improve the robustness of the network.
2. Intensity calibration: The intensity calibration is applied to multispectral images at different wavelengths to obtain the spectral profile that is close to the final recovered image. The variation in intensity is caused by the small vibration of the light source and the transmitted deviation of different wavelength channels.
3. Denoising: Noise, an important factor of diffractive multispectral imaging, causes the difference between simulated and measured information, which includes environmental noise, dark current, photon noise, readout noise, and analog-to-digital converter (ADC) noise. Google’s MAXIM model is used as the preprocessor to remove the noise of the aliased images.
4. 3D U-Net: Calibrated diffractive multispectral images are trained by the 3D U-Net to reconstitute multispectral images. The 3D U-Net network is composed of the encoding module and decoding module based on the U-Net framework. The encoder consists of a down-sampling and feature extraction module that transforms the input 3D multispectral image into a multichannel feature map. Also, the decoding module with an up-sampling and image reconstruction module reduces the multichannel 4D feature tensor to the 3D multispectral image. Both feature extraction modules and image reconstruction modules are made up of a norm layer, 3D convolution layer, rectified linear unit (ReLU), and simplified channel attention (SCA) layer. The 3D convolution layer and the SCA layer are utilized to capture 3D features and adjust the weight between adjacent spectral channels to reconstruct diffractive multispectral images.

The aforementioned network structure was implemented by using python programs. Numerical experiments were conducted on a server with an Intel(R) Xeon(R) Platinum 8255C CPU and two RTX 3080 (10 GB) GPUs. The operating system for the experiment environment was Ubuntu 20.04.4 LTS. The model was constructed through the pytorch framework, version 1.11.0, and was guided by the open-source BasicSR library [24]. In this way, our HSC3D algorithm combines the advantages of the space calibrated module and 3D U-Net, which is able to learn spatial and intensity calibrated factors and obtain network parameters automatically.

## 3. Diffractive Multispectral Imaging System

**Figure 3:**
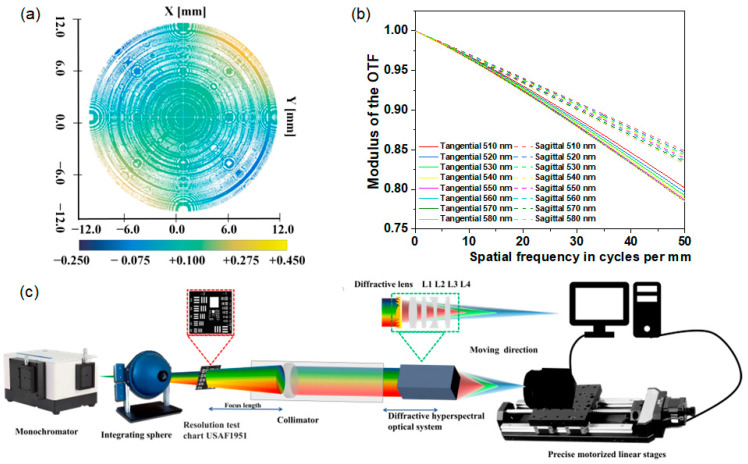
(**a**) The measured microcosmic appearance of the fabricated diffractive lens. (**b**) The simulated MTF of the diffractive multispectral optical imaging system for a wavelength ranging from 510 nm to 580 nm, where the solid line and the dashed line depict the tangential and sagittal results, respectively. (**c**) The experimental schematic for the diffractive multispectral optical imaging system.

The diffractive multispectral optical imaging system is designed and fabricated to obtain multispectral information. As shown in Figure 3, the experimental diffraction multispectral imaging system consists of one diffractive lens and four refractive lenses, whose monochromatic aberration has been carefully corrected to achieve high-quality imaging. The diffractive lens is fabricated by photolithography, and its microcosmic appearance is measured by LuphoScan50 with an accuracy of 2 nm by using a non-contact scanning method. The measured microcosmic appearance of the fabricated diffractive lens in Figure 3a shows the deviation of the fabricated diffractive lens, which is around 78.89% less than 0.1 um, satisfying the wave aberration caused by the above deviation errors of less than 1/10λ. From Figure 3b, the simulated modulation transfer function (MTF) of our diffractive multispectral optical imaging system is higher than 0.76 for the wavelength ranging from 510 to 580 nm, demonstrating the high imaging quality of our diffractive multispectral optical imaging system.

The experimental schematic for the diffractive hyperspectral optical imaging system is shown in Figure 3c. The uniformly illuminated incident light from the monochromator (Omno150300) and integrating sphere (NBT-JF-150m) passed through the USAF1951 calibration target and carried the spatial information of different spectral images. Then, the transmitted light traveled through the collimator with a focus of 500 mm at 555 nm and formed parallel light. Further, this parallel light was input into the fabricated diffractive multispectral imaging system. Finally, the light intensity of the diffractive multispectral channels was recorded by moving the complementary metal-oxide semiconductor (CMOS) pixel sensor installed on the stepper motors (Zolix PA300), with a moving accuracy of around 25 μm. During the experimental process, monochromatic light with an interval of 10 nm was used to calibrate the focus position for different wavelengths. In addition, wide-spectrum light was used to record the actual images. A 550 nm filter (EO65744) with a spectral width of 80 nm was inserted between the monochromator and integrating sphere to prevent the interference caused by outside light.

## 4. Diffractive Multispectral Imaging Experimental Results

**Figure 4:**
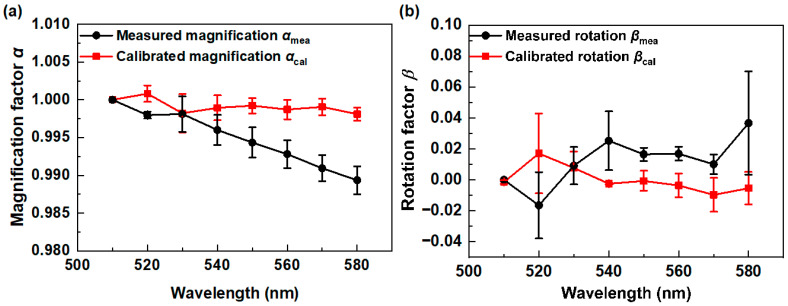
(**a**) The magnification factors *α* and (**b**) rotation factors *β* of the 8-channel multispectral images, where the black line and red line depict the measured and calibrated results, respectively.

Magnification factors *α* and rotation factors *β* are computed by using the intensity-based automatic image registration methods. The features of eight-channel images are identified by the Gaussian differential function, where each feature point possesses three messages including position, scale, and direction. Also, the geometric transform matrix of eight-channel images is calculated by fitting the paired feature points using the least squares method. From Figure 4a, the measured magnification factor *α*_mea_ (black line) declines with the increasing wavelength along the optical axis with a larger focus distance of the imaging system. The calibrated magnification factor *α*_cal_ (red line) in Figure 4a is calculated by using the image warping algorithm and is closer to 1, presenting a smaller deviation compared to the measured magnification factor *α*_mea_ (black line). In addition, the measured random rotation factor *β*_opt_ in Figure 4b (black line) is corrected by using the image warping algorithm, and the calibrated random rotation factor *β*_opt_ (red line) is closer to 0° in the wavelength of 570 nm and 580 nm. In addition, the measurement errors in the magnification factor *a*_mea_ and the rotation factor *b*_mea_ are around 0.0015 and 0.0126, respectively, which can be attributed to the effect of different measurement backgrounds during multispectral diffractive imaging. Furthermore, the calibration errors of the magnification factor *a*_cal_ and the rotation factor *b*_cal_ are around 0.0012 and 0.0092, respectively, which can arise from the data processing of the calibration model. As a result, the proposed measurement and calibration errors have quite a small value of around 0.01, confirming that our proposed calibrated module could decrease the space-variant issues.

Furthermore, the MPSNR and MSSIM parameters are calculated to characterize the effectiveness of space calibration and the imaging quality of the multispectral images. The peak signal-to-noise ratio (PSNR) typically assesses the similarity between the two images and satisfies the following [25]:

(8) $$PSNR=20\log_{10}\left(\frac{\max\left(I\right)}{\sqrt{MSE}}\right).$$

where the mean squared error (MSE) can be calculated by

(9) $$MSE=\frac{1}{mn}\sum_{i=0}^{m-1}\sum_{j=0}^{n-1}∥I\left(i,j\right)-K\left(i,j\right){∥}^{2}.$$

Also, the structure similarity index measure (SSIM) depicts the effect of brightness, contrast, and structure, and is consistent with the subjective perception of image quality by the human eye [26], which can be calculated as follows:

(10) $$SSIM\left(x,y\right)=\frac{\left(2\mu_{x}\mu_{y}+C_{1}\right)\left(2\sigma_{xy}+C_{2}\right)}{\left({\mu_{x}}^{2}+{\mu_{y}}^{2}+C_{1}\right)\left({\sigma_{x}}^{2}+{\sigma_{y}}^{2}+C_{2}\right)}$$

where $\mu_{x}$, $\sigma_{x}$, and $\sigma_{xy}$ can be calculated by the following:

(11) $$\begin{array}{c} \mu_{x}=\sum_{i=1}^{N}w_{i}x_{i} \end{array}$$

(12) $$\sigma_{x}=(\sum_{i=1}^{N}w_{i}(x_{i}-\mu_{x}{)}^{2}{)}^{\frac{1}{2}}$$

(13) $$\begin{array}{c} \sigma_{xy}=\sum_{i=1}^{N}w_{i}(x_{i}-\mu_{x})(y_{i}-\mu_{y}) \end{array}$$

As can be seen from Table 1, the calculated PSNR values of the multispectral channel images at wavelengths of 520 nm, 530 nm, 540 nm, 550 nm, 560 nm, 570 nm, and 580 nm are 9.95 dB, 10.89 dB, 11.93 dB, 12.71 dB, 13.11 dB, 13.26 dB, and 13.36 dB, respectively, which are higher than the original values. Similarly, the calculated SSIM values of the multispectral channel images at wavelengths of 520 nm, 530 nm, 540 nm, 550 nm, 560 nm, 570 nm, and 580 nm are 0.53, 0.57, 0.64, 0.67, 0.62, 0.65, and 0.68, respectively, which are also higher than the original images. In order to fully evaluate the multispectral imaging performance, the values of the MPSNR and MSSIM are also calculated. In comparison with that of the original eight-channel multispectral images, the MPSNR and MSSIM values are improved by 0.11 dB and 0.04, respectively. As a result, our calibration model shows good performance in reducing the space-variant issues of magnification and rotation effects and increasing the imaging quality of diffractive multispectral imaging.

**Figure 5:**
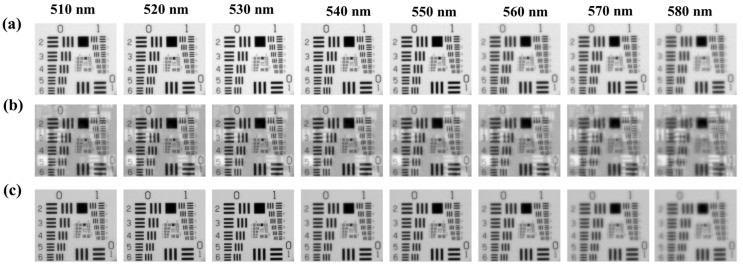
Reconstructed 8-channel (510 nm, 520 nm, 530 nm, 540 nm, 550 nm,560 nm, 570 nm, and 580 nm) multispectral images from (**a**) 3D U-net, (**b**) Unrolled Network, and (**c**) our HSC3D network.

The calibrated multispectral images are further input into 3D U-Net to reconstruct the multispectral images. Figure 5a–c show the reconstructed eight-channel multispectral images by using the 3D U-Net, Unrolled Network algorithm, and our HSC3D network, respectively. As shown in Figure 5a, the imaging quality of the simulated reconstructed eight-channel multispectral images by using the 3D U-Net algorithm is obviously decreased when the wavelength is increased, especially at 580 nm. This trend may come from the magnification and rotation effects shown in Figure 4, which is also in accordance with reported research that describes similar decreased imaging quality properties [22,23]. The reconstructed eight-channel multispectral images by using the Unrolled Network in Figure 5b present the typical ghosting issue that multispectral images erroneously retain, and also exhibit a similar decreasing imaging quality effect to that in Figure 5a. The reconstructed spectral images obtained by using our HSC3D network (Figure 5c) are clearer than the reconstructed spectral images in Figure 5a and avoid the ghosting issues present in Figure 5b. Further, the PSNR and SSIM of the Unrolled Network and our HSC3D network are calculated and analyzed to characterize the imaging performance quantitatively.

As shown in Table 2, the calculated values of the PSNR of the eight-channel recovered images by using our HSC3D network at wavelengths of 510 nm, 520 nm, 530 nm, 540 nm, 550 nm, 560 nm, 570 nm, and 580 nm are 8.90 dB, 9.64 dB, 10.44 dB, 11.19 dB, 11.85 dB, 12.41 dB, 12.84 dB, and 13.00 dB, respectively, which are larger than those obtained by using the Unrolled Network. Similarly, the calculated values of the SSIM of the eight-channel recovered images by using our HSC3D network are 0.54, 0.58, 0.63, 0.65, 0.65, 0.64, 0.65, and 0.65, respectively, which are also higher than those obtained by using the Unrolled Network. These improvements in the PSNR and SSIM of the eight-channel recovered images are in accordance with Figure 5b,c, confirming an improved imaging quality compared with the Unrolled Network. Furthermore, the calculated values of MPSNR_hyb_ and MSSIM_hyb_ of the eight-channel recovered images by using our HSC3D network are around 11.28 dB and 0.62, respectively, which are 3.33 dB and 0.08 higher than those obtained by using the Unrolled Network, respectively. All of these improvements show that our new algorithm has a better performance in terms of overall spectral image restoration results.

## 5. Conclusions

In conclusion, we proposed a novel high-quality and efficient hybrid space calibrated 3D Network “HSC3D” for space-variant diffractive multispectral imaging. Our “HSC3D” network utilizes the 3D U-Net structure combined with space calibration models of magnification and rotation correction to achieve more accurate multispectral restoration. A prototype diffractive multispectral imaging system was designed and manufactured which consisted of one diffractive lens and four refractive lenses, whose monochromatic aberration is corrected carefully to realize high-quality multispectral imaging. The measured eight-channel multispectral images with variant space effects of magnification and rotation were calibrated by employing an intensity-based automatic image registration module, and were then input into the 3D network to reconstruct multispectral images. The calculated values of the MPSNR and MSSIM of the reconstructed eight-channel multispectral images obtained by using our hybrid space calibrated 3D network are shown to be improved by 3.33 dB and 0.08, respectively, in comparison with the original ones, confirming the obviously improved image quality in comparison with the typical Unrolled Network algorithm. Our algorithm combines the advantages of the space calibration model and U-Net architecture with 3D convolutional layers to improve the image quality of diffractive multispectral imaging, and thus has no need for large amounts of experimental data and complex equipment modifications. More complex physical variation effects of the PSF, such as the aberrations of different optical elements, can also be discussed and calibrated in future work to achieve an even wider spectrum and more sensitive diffractive multispectral imaging capability. Moreover, a discrete pixel-by-pixel PSF of the diffractive multispectral imaging array can also be researched to advance gazing diffractive multispectral imaging by using a grid hybrid space calibrated 3D algorithm. The proposed HSC3D network can also be adapted to more complex practical cases, especially for cases with various spatial variants such as space imaging [27], micrography [28], and security camera systems [29].

## Figures and Tables

**Table 1:** Comparison of PSNR and SSIM between calibrated and original 8-channel multispectral images.

Wavelength (nm)	PSNRmea	SSIMmea	PSNRcal	SSIMcal
510.00	9.09	0.48	9.09	0.48
520.00	9.84	0.51	9.95	0.53
530.00	10.77	0.55	10.89	0.57
540.00	11.80	0.60	11.93	0.64
550.00	12.58	0.63	12.71	0.67
560.00	12.97	0.61	13.11	0.62
570.00	13.15	0.60	13.26	0.65
580.00	13.25	0.60	13.36	0.68
Mean value	11.68	0.57	11.79	0.61

**Table 2:** Comparison of PSNR and SSIM between Unrolled Network and our HSC3D network algorithms.

Wavelength (nm)	PSNRhyb	SSIMhyb	PSNRunr	SSIMunr
510.00	4.03	0.44	8.90	0.54
520.00	7.87	0.47	9.64	0.58
530.00	8.66	0.52	10.44	0.63
540.00	9.08	0.53	11.19	0.65
550.00	9.08	0.62	11.85	0.65
560.00	8.70	0.60	12.41	0.64
570.00	8.19	0.57	12.84	0.65
580.00	7.97	0.54	13.00	0.65
Mean value	7.95	0.54	11.28	0.62

## Data Availability

Data can be obtained from the authors upon reasonable request.
